# Substrate Recognition Analysis in an Ethyl Carbamate-Hydrolyzing Esterase and Semi-Rational Baijiu Flavor Preservation

**DOI:** 10.3390/foods15173118

**Published:** 2026-09-02

**Authors:** Binchen Wang, Naihui Dong, Siyu Xue, Yuxuanwen Zhao, Chaofan Ji, Beiwei Zhu, Sufang Zhang

**Affiliations:** 1 SKL of Marine Food Processing & Safety Control, National Engineering Research Center of Seafood, Collaborative Innovation Center of Seafood Deep Processing, School of Food Science and Technology, Dalian Polytechnic University, Dalian 116034, China; wbc4122@163.com (B.W.); dongnaihui98@163.com (N.D.); morganxue@126.com (S.X.); hsuanvane@163.com (Y.Z.); jicf@dlpu.edu.cn (C.J.); 2 Analysis and Test Center, Chinese Academy of Tropical Agricultural Sciences, Haikou 571101, China; 3 School of Chemical Engineering, University of Science and Technology Liaoning, Anshan 114051, China

**Keywords:** ethyl carbamate, semi-rational design, molecular dynamics, substrate specificity, Chinese Baijiu

## Abstract

Ethyl carbamate (EC) is a widely recognized carcinogen present in fermented foods, and enzymatic degradation is a safe and effective strategy for its reduction. An EC-degrading esterase derived from *Acinetobacter calcoaceticus* (AcECH) was previously identified. However, volatile ester compounds in Chinese Baijiu compete with EC for enzyme binding, thereby affecting degradation efficiency and flavor quality. In this study, molecular docking, molecular dynamics (MD) simulations, enzyme kinetics, bio-layer interferometry (BLI), and gas chromatography–ion mobility spectrometry (GC-IMS) were employed to investigate the substrate specificity of AcECH. Based on computational prediction and experimental validation, F157G has been identified as the most suitable mutant for improving substrate recognition. Compared with the wild-type enzyme, the degradation rates of ethyl acetate, ethyl valerate, and ethyl hexanoate decreased to 64.23%, 49.28%, and 34.08%, respectively. Meanwhile, F157G showed enhanced apparent binding affinity toward EC while largely maintaining EC hydrolytic activity, and reduced the degradation of desirable volatile esters, indicating an altered substrate preference of AcECH.

## 1. Introduction

Ethyl carbamate (EC), classified as a Group 2A carcinogen by the International Agency for Research on Cancer (IARC), is naturally generated during fermentation through the reaction of ethanol with nitrogen-containing precursors (such as urea, cyanate, and carbamyl compounds) and is widely detected in various fermented foods [1]. Prolonged consumption of foods with high EC content poses significant risks to human health [2]. The World Health Organization (WHO) and numerous countries worldwide have set strict regulatory limits for EC in fermented foods [3]. Given the chemical stability of EC after formation, strategies based on reducing precursor accumulation or modifying fermentation parameters often show limited effectiveness and may negatively influence the sensory properties of fermented products. Consequently, enzymatic degradation has attracted increasing attention because of its specificity, mild reaction conditions, and compatibility with food processing systems. Compared with traditional physical or chemical methods as well as microbial methods, enzymatic hydrolysis offers a promising approach to reducing the EC level, while minimizing the adverse effects of other methods on the overall quality of the product [4].

Enzymatic strategies for EC mitigation mainly involve two approaches: reducing the accumulation of EC precursors and directly degrading the formed EC. In recent years, several microbial-derived EC-degrading enzymes have been reported, including urethanases and esterase-type EC hydrolases. These enzymes can directly hydrolyze EC into ethanol, carbon dioxide, and ammonia (or ammonium ions), providing a promising approach for eliminating pre-existing EC in fermented foods. Previous studies have demonstrated that EC-degrading enzymes from microorganisms such as *Acidisoma silvae* and *Lichtheimia ramosa* can effectively reduce EC levels in fermented beverages, and other fermentation-based foods [5,6]. Moreover, recently reported salt-tolerant urethanases have further improved the potential application of EC-degrading enzymes in complex food matrices [7].

However, many reported EC-hydrolyzing enzymes are amidase-like enzymes that can act on both EC and its precursor urea. The high concentration of urea commonly present in fermentation systems may result in substrate competition, thereby limiting their ability to efficiently degrade the EC that has already accumulated [8,9,10]. Consequently, esterase-type EC hydrolases capable of directly targeting the carbamate bond of EC have attracted increasing attention. At the same time, current studies have mainly focused on improving EC degradation efficiency, enzymatic properties, and environmental tolerance, whereas the molecular mechanisms underlying substrate discrimination, particularly the ability of EC-degrading enzymes to distinguish EC from structurally similar flavor esters and minimize undesirable flavor loss during EC removal, remain poorly understood.

In the previous study, an esterase, designated AcECH, was isolated, demonstrating superior EC degradation capabilities. This enzyme specifically targets the carbamate ester bond within the EC structure and exhibits no hydrolytic activity towards urea, thereby effectively mitigating the issue of substrate competition [11]. However, comparative analyses utilizing gas chromatography-mass spectrometry (GC-MS) revealed that AcECH also interacts with volatile ethyl ester compounds containing ester bonds, such as ethyl acetate, ethyl hexanoate, and ethyl pentanoate. These ethyl esters are recognized as significant contributors to the desirable flavor profile of Chinese Baijiu. Therefore, it is necessary to explore the molecular mechanism of the catalytic effect of AcECH on EC, ethyl acetate, ethyl valerate and ethyl hexanoate, which can provide a theoretical basis for the optimization of the substrate specificity of AcECH in the future.

To thoroughly explore the catalytic mechanisms of AcECH and facilitate its rational modification, a semi-rational design strategy was employed in this study. Molecular dynamics (MD) simulations were initially performed to identify key amino acid residues potentially affecting the enzymatic properties, which were subsequently validated through site-directed mutagenesis. The integration of molecular docking and MD simulations has been widely applied to investigate enzyme–ligand interactions and identify critical residues involved in substrate recognition and catalytic processes [1,12,13]. This computational strategy provides valuable insights into the structural basis of enzyme function and facilitates the targeted engineering of enzyme specificity and stability [14].

In summary, in this study, the molecular basis underlying substrate recognition of AcECH toward EC was investigated using molecular simulation and experimental approaches. The degradation effects and binding affinities of AcECH on several characteristic volatile compounds present in Chinese Baijiu—namely, ethyl acetate, ethyl valerate and ethyl hexanoate, were comparatively analyzed before and after treatment using GC-IMS and BLI. Finally, the binding modes and binding energies of AcECH with the three volatiles were calculated and compared to those with EC, thereby elucidating the molecular-level binding differences of AcECH to the four ligands and identifying the key amino acids that influence the substrate specificity of AcECH. Based on these mechanistic insights, targeted modifications were introduced via site-directed mutagenesis to retain catalytic efficiency towards EC while effectively suppressing degradation efficiency towards ethyl acetate, ethyl valerate, and ethyl hexanoate, thereby enhancing substrate specificity.

## 2. Materials and Methods

### 2.1. Bacterial Strains and Chemicals

Bacterial Strains: The strain Escherichia coli BL21(DE3)/pET24-AcECH is maintained in the laboratory.

Chemicals: Ethyl acetate (GC, ≥99.9%), ethyl valerate (≥98%), and ethyl hexanoate (≥99%) were purchased from Shanghai Aladdin Biochemical Technology Co., Ltd. (Shanghai, China). Ethyl carbamate (98%) and DMSO (99%) were obtained from Shanghai Macklin Biochemical Technology Co., Ltd. (Shanghai, China). C4-C9 ketones (GC, 10 ppm each component) were sourced from Gesellschaft für analytische Sensorsysteme mbH (Dortmund, Deutschland). The inducer isopropyl-beta-D-thiogalactopyranoside (IPTG) was purchased from Shanghai Aladdin Biochemical Technology Co., Ltd. (Shanghai, China). 1 × PBS (pH7.2–7.4) and Tween 20 were purchased from Beijing Solarbio Science & Technology Co., Ltd. (Beijing, China). Octet^®^ Streptavidin (SA) Biosensors were purchased from Sartorius AG (Gottingen, Germany). The biotinylation kit was purchased from Beijing Science LC Technology Co., Ltd. (Beijing, China). The PrimeSTAR HS DNA Polymerase and Dpn1 was purchased from Takara Biomedical Technology Co., Ltd. (Beijing, China). Luria–Bertani (LB) broth supplied by Hope Bio-Technology Co. (Qingdao, China) contained 1% tryptone (*w*/*v*), 0.5% yeast extract (*w*/*v*), 1% NaCl (*w*/*v*), with a pH of 7.0. Kanamycin (Kan) sulfate was purchased from Sangon Biotech (Shanghai, China). Bradford Protein Assay Kit was obtained from Beyotime Biotechnology (Shanghai, China). Primer synthesis and DNA sequencing were completed using Sangon Biotech Co., Ltd. (Shanghai, China).

### 2.2. Expression, Purification and SDS-PAGE Analysis of AcECH

The strain *E. coli* BL21(DE3)/pET24-AcECH was constructed by Dong Naihui [11]. After activation on LB agar plate supplemented with 50 μg/mL kanamycin, single colonies were inoculated into 10 mL of LB broth containing kanamycin (50 μg/mL) and incubated for 12 h at 37 °C. Subsequently, a 2% inoculum was transferred to 400 mL of the same medium and incubated at 37 °C with shaking at 200 rpm. When the OD_600_ reached 0.6, IPTG was added to a final concentration of 1 mM and incubated at 20 °C for another 20 h. Bacterial pellets were collected by centrifugation at 4000 rpm for 20 min, resuspended in phosphate buffer (pH 8.0), and disrupted by ultrasound (400 watts, 3 s on, 5 s off). The supernatant of the lysate was prepared by centrifugation at 4000 rpm for 20 min. Subsequently, the target protein was purified using nickel column chromatography. AcECH was eluted with an elution buffer consisting of 20 mM sodium phosphate, 500 mM NaCl, and 300 mM imidazole (pH 7.4). The target proteins were then desalted using ultrafiltration.

### 2.3. Enzyme Activity Assay and Kinetic Analysis

Since the enzymatic hydrolysis by EC directly produces ammonia, the phenol-chlorite colorimetric method is used to quantitatively determine the AcECH activity based on the amount of ammonium ions released. This method can directly reflect the hydrolysis ability of the enzyme by EC. Termination solution: Trichloroacetic acid (10 g) was dissolved in ultrapure water, and the volume was adjusted to 100 mL. Chromogenic reagent I: Phenol (15 g) and sodium nitroprusside (0.625 g) were dissolved in ultrapure water, and the final volume was adjusted to 250 mL. Chromogenic reagent II: Sodium hydroxide (13.125 g) and sodium hypochlorite solution (7.5 mL) were dissolved in ultrapure water, and the volume was adjusted to 250 mL. Appropriately diluted enzyme solution (50 μL) was mixed with 50 μL of EC solution in PBS buffer and incubated at 70 °C for 15 min. The reaction was terminated by adding 100 μL of stop solution, followed by the sequential addition of 100 μL of color reagent I and 100 μL of color reagent II. After incubation at 37 °C for 15 min, the absorbance at 625 nm was measured using a microplate reader. The amount of ammonia produced was quantified using an ammonium standard curve (as shown in Figure S1). One unit (U) of AcECH activity was defined as the amount of enzyme that released 1 μmol of ammonia per minute under the assay conditions.

For kinetic analysis, EC solutions were prepared and mixed with 50 μL of appropriately diluted AcECH solution (0.3048 mg/mL) at a 1:1 (*v*/*v*) ratio. The final EC concentrations in the reaction mixtures were therefore 1.25–800 mM. Accordingly, the molar ratio of EC to AcECH ranged from approximately 1240 to 159,000, indicating that the substrate concentration was sufficiently higher than the enzyme concentration for Michaelis–Menten analysis. Reactions were conducted under the conditions described above for 5 min, and the amount of ammonia produced was quantified using the aforementioned colorimetric assay. Initial reaction rates were calculated from the amount of ammonia generated and fitted to the Michaelis–Menten equation by nonlinear regression using GraphPad Prism 10 (GraphPad Software, San Diego, CA, USA) to determine the apparent Km and Vmax values.

### 2.4. Collection of Volatile Compounds After Enzyme Treatment and the GC–IMS Analysis

Ethyl acetate, ethyl valerate, and ethyl hexanoate were diluted to appropriate concentrations, respectively. A total of 100 μL of each substrate solution was combined with 100 μL of pure enzyme solution (solutions were adjusted to the same protein concentration of 0.19 mg/mL) and mixed thoroughly. Then the reaction was allowed to proceed for 12 h at 30 °C. Subsequently, 50 μL of the reaction sample was transferred into a 20 mL headspace vial, which was then sealed. The vial was incubated at 60 °C for 10 min to stabilize the air within. Following this, 500 μL of headspace gas was injected into the GC-IMS injection port, maintained at 80 °C, through the upper sample needle, which was set at 85 °C. The volatile organic compounds were propelled through the carrier gas (N_2_, purity ≥ 99.999%) into the GC column for analysis at 40 °C. The carrier gas program was as follows: 2 mL/min for 2 min; up to 15 mL/min for 8 min; then up to 100 mL/min, which was then maintained for an additional 10 min. The total run time for the GC analysis was 30 min. The flow rate of the drift gas (N_2_, purity ≥ 99.999%) was set at 150 mL/min. An average of 12 scans was performed for each spectrum. In order to normalize the retention properties of individual GCs to the retention index library, instrumental analyses were performed on the stock solutions of mixed standards under the same method to calculate the retention indices (RIs) of the VOCs and establish the standard curves to characterize the volatiles. The degradation efficiency was normalized according to the amount of enzyme added and expressed as substrate degradation rate per milligram of enzyme (%).

### 2.5. AcECH Modeling

**Figure 1:**
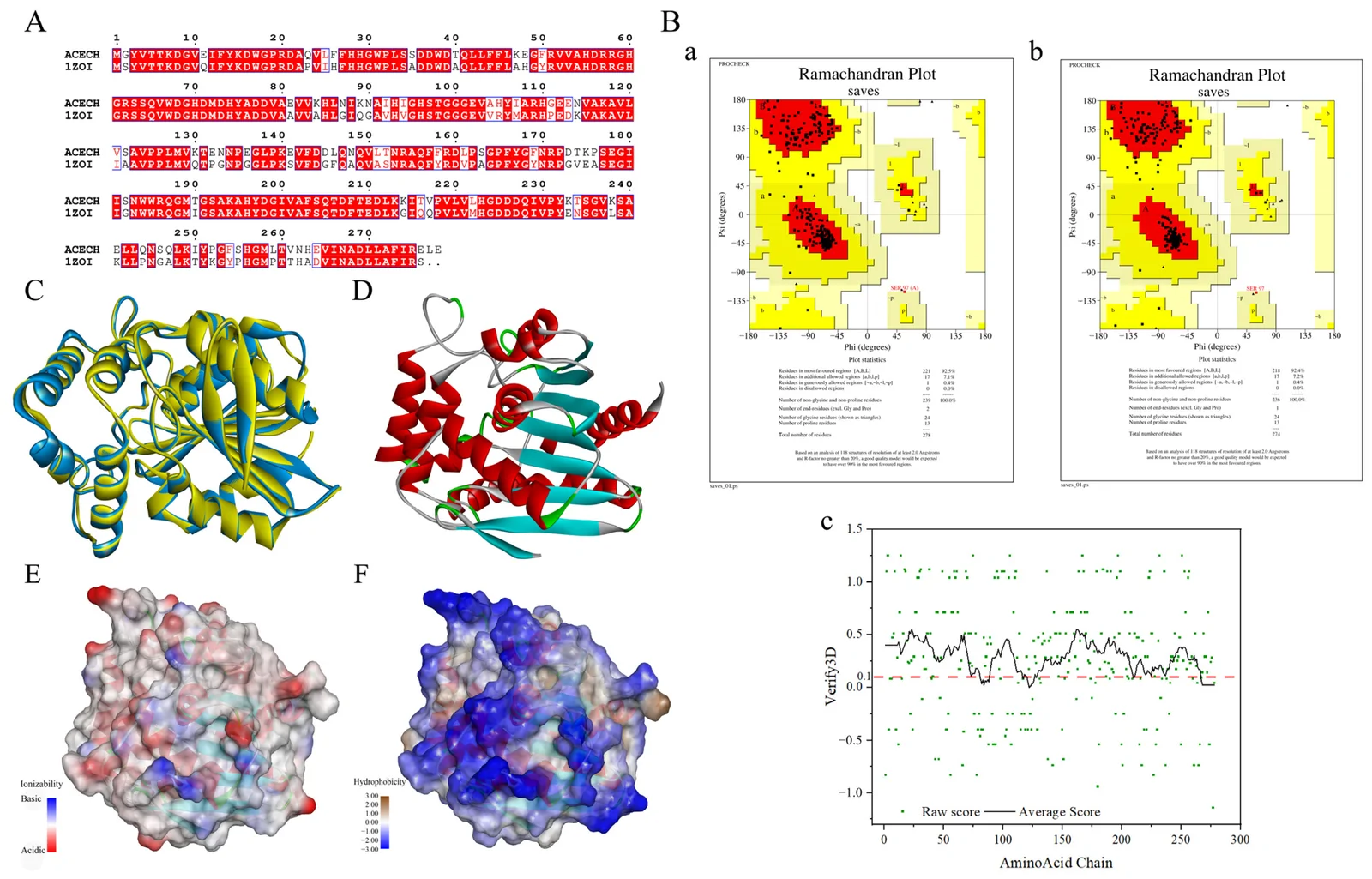
Modeling of AcECH. (**A**) Sequence alignment of AcECH with template protein (PDB ID: 1ZOI); (**B**) (**a**) The Ramachandran Plot of DS mode. Red, yellow, pale yellow, and white regions represent the most favored, additionally allowed, generously allowed, and disallowed regions, respectively; (**b**) and The Ramachandran Plot of AF3 model; (**c**) Verify3D of AF3 model; (**C**) Overlay of DS model and AF3 model; (**D**) 3D structure of AF3 model; (**E**) Surface charge distribution of AcECH; (**F**) Surface hydrophobicity of AcECH.

Prior to constructing the receptor model by Discovery Studio 2017 R2 software (DS, Dassault Systemes Biovia, San Diego, CA, USA), proteins with high amino acid sequence similarity to the receptor were identified through comparison in the Protein Data Bank (PDB) library. The crystal structure of the template protein annotated as esterase (PDB ID: 1ZOI) was obtained with 76% sequence similarity (Figure 1A). The structure of this protein after removing unnecessary ligands and optimized for hydrogenation was used as a template to build 20 AcECH models, and the model with the highest confidence was selected as the DS modeling result using PDF Total Energy and DOPE score as the measures. Subsequently, the most credible results were obtained by uploading the amino acid sequences of AcECH in the Alphafold 3 (AF3) online AI calculation tool (DeepMind Technologies Limited, London, UK). The dynamic simulation process was performed using the CHARMM 36 force field [15,16]. In order to verify the reliability of the models and to compare the quality and credibility of the AF3 and DS models, the constructed models were uploaded to the SVAES protein online assessment website (UCLA-DOE Institute, Los Angeles, CA, USA), and the quality assessment was performed using Procheck [17,18] and Verify3D [19,20] (Figure 1B), and the model with the highest overall credibility was selected for subsequent studies.

### 2.6. Molecular Docking

The binding of AcECH with EC and ethyl hexanoate, ethyl valerate and ethyl acetate was analyzed using a molecular docking method. Ligand molecules such as EC were modeled in DS software, followed by energy minimization to obtain ligand molecular structures with high confidence. The Flexible Docking system in DS was used for the molecular docking of the receptor and ligand, allowing for flexibility in both the active site of the protein and the conformation of the ligand, thereby accommodating potential binding modes. Prior to docking, the AF3 model underwent a Prepare Protein treatment, which included atom name normalization and other preprocessing steps. The active site of AcECH was determined based on the active site of the template protein, and the volume of the receptor cavity was set as a flexible region; then the ligand was subjected to multiple interactions with this region. Multiple binding poses were evaluated based on docking energy, and the optimal docking pose was preserved.

### 2.7. Molecular Dynamics Simulations

After docking, the optimal docking pose was solvated under the Charmm 36 force field to create an aqueous cubic box of protein–ligand complexes with water as solvent. The solvent system includes 6115 water molecules (TIP), 23 sodium ions, and 16 chloride ions. The established system was first subjected to energy minimization, followed by three phases of simulated annealing, equilibrium and sampling. Finally, the simulated process trajectory was analyzed. The simulation parameters were set as follows: the temperature was gradually increased from 50 K to 300 K, and the simulation time was 50 ps, of which 20 ps was for the equilibrium stage and 200 ps for the sampling stage. Root Mean Square Deviation (RMSD) and Root Mean Square Fluctuation (RMSF) were calculated by the module that comes with the DS program to describe the dynamic trajectory of the process.

### 2.8. Conservative Sequence Analysis

To evaluate the evolutionary conservation of the selected residues, homologous protein sequences were retrieved from the NCBI database using BLAST (version 2.17.0, Bethesda, MD, USA). Multiple sequence alignment was performed using MAFFT (Kyoto University, Tokyo, Japan), and residue conservation was visualized using Jalview (University of Dundee, Dundee, UK). The conservation scores were further analyzed based on the aligned homologous sequences.

### 2.9. Construction of Mutants

The mutant was obtained using whole-plasmid PCR. The reaction program was as follows: 1 cycle of 180 s at 94 °C, 30 cycles of 10 s at 94 °C, 300 s at 68 °C, and 1 cycle of 10 min at 72 °C. After digestion by Dpn I at 37 °C for 1 h, the PCR products were introduced into *E. coli* BL21(DE3) for cloning. The correct variant genes were confirmed by DNA sequencing. The mutants were constructed as described above, using pET24-AcECH as the template; the primers are shown in Table S1.

### 2.10. Bio-Layer Interferometry Analysis of Substrate Binding Affinity

Purified wild-type AcECH and mutant F157G proteins were diluted in phosphate-buffered saline (PBS, pH 7.4) containing 0.02% Tween20 and 5% DMSO. SA biosensors were hydrated in buffer solution for 10 min prior to use and subsequently loaded with proteins until a stable baseline signal (2.5 nm) was obtained. Following protein immobilization, the biosensors were equilibrated in buffer for 60 s and then transferred into substrate solutions containing different concentrations of ligands such as EC for binding analysis. The association step was monitored for 120 s, followed by dissociation in substrate-free buffer for an additional 120 s. All experiments were conducted at 30 °C with continuous shaking at 1000 rpm. The obtained sensorgrams were fitted using a 1:1 binding model provided by the Octet Data Analysis software version 14.

### 2.11. Statistical Analysis

All experiments were independently performed in triplicate, and the data are presented as mean ± standard deviation (SD). The SD values were calculated based on independent experimental replicates to evaluate measurement variability. The consistency among replicates was examined before further analysis, and no experimental data were excluded unless technical errors occurred during the procedure. Statistical analyses and graphical representations were performed using Origin 2019 software (OriginLab Corp., Northampton, MA, USA).

## 3. Results

### 3.1. Construction of AcECH 3D Model

Currently, AcECH does not possess an experimentally determined accurate 3D structure. Therefore, the simulated 3D structure of AcECH was modeled using DS software through a homology modeling approach, as well as AF3 [21]. In order to visualize the differences between the constructed models obtained by the two methods, the models were superimposed and compared using DS (Figure 1C). It was found that the main tertiary structure, as well as the central core region of both models, exhibited high similarity and overlap, with variability primarily occurring in the irregular loop regions at the periphery of the structures. Consequently, the two model construction methods did not significantly influence the predictions regarding protein stability and function. Subsequently, the two models were separately subjected to Ramachandran plot analysis to compare the model quality (Figure 1B(a,b)) [22]. The proportion of amino acid residues in the most favorable and permissive regions of the entire protein was 99.6%, exceeding 90% for both models. This indicates that the two model conformations comply with the stereochemical rules and are acceptable for further kinetic analyses. The percentage of amino acids located in the most favorable region of the AF3 model was 92.5%, slightly higher than the 92.4% observed for the DS model. The compatibility between the atomic model (3D) and the corresponding amino acid sequence (1D) was determined using the Verify3D software SAVES V6.1 (Figure 1B(c)). Over 89.57% of the residues had an average 3D-1D score of ≥0.1 in the AF3 model, indicating that the quality of this model is very high. Therefore, the protein structure simulated by AF3 was selected for further analysis in the subsequent study (Figure 1D).

To further elucidate the mechanism of action and stability of the enzyme, the surface charge distribution and hydrophobicity of AcECH were analyzed by DS software (Figure 1E,F). The analysis revealed that the protein surface is more hydrophilic, suggesting enhanced water solubility. This phenomenon is consistent with the inherent properties of the protein, and the presence of hydrophilic regions facilitates better binding and interaction with ligand molecules in the surrounding solvent environment.

### 3.2. Molecular Docking of AcECH with EC

**Figure 2:**
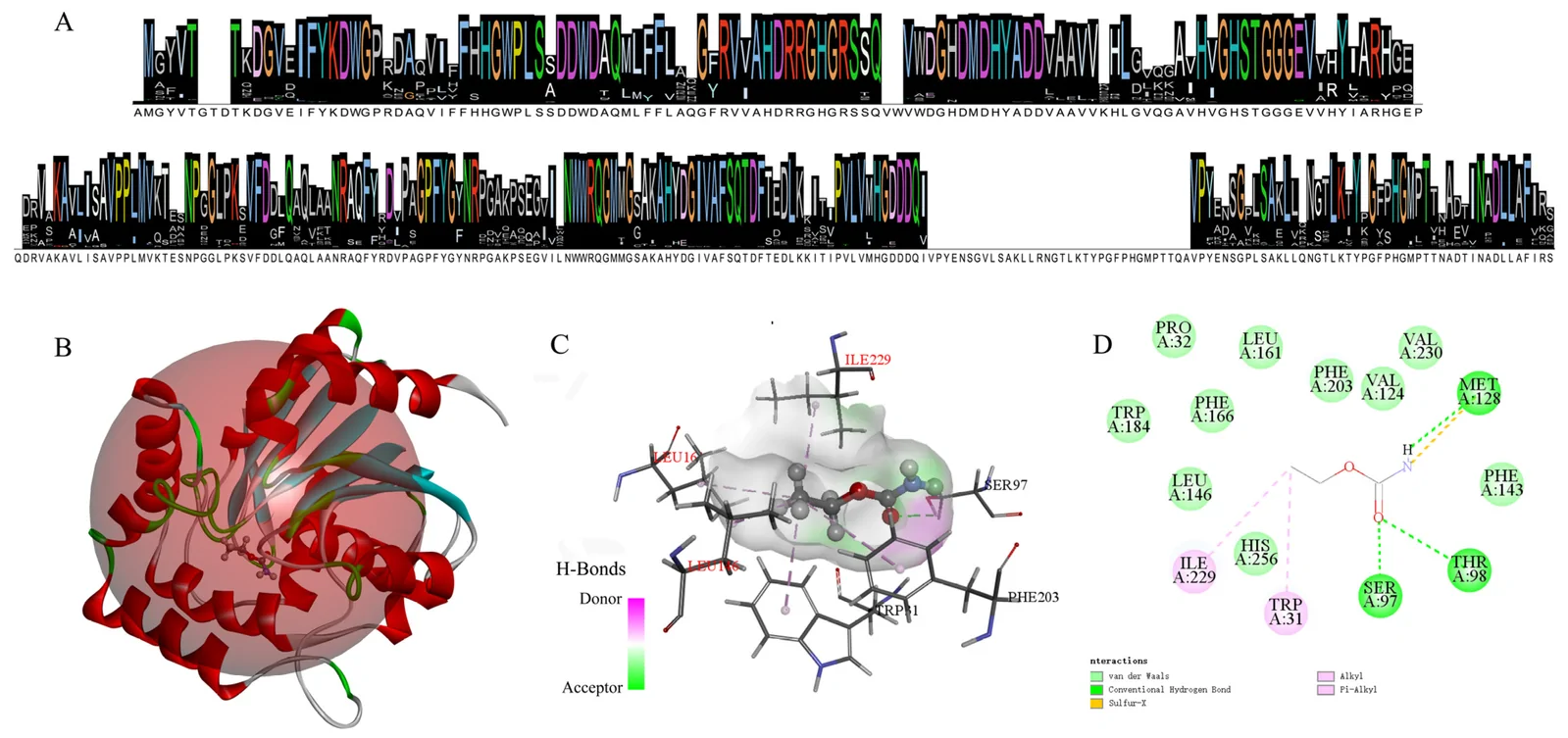
(**A**) Evolutionary conservation of AcECH. (**B**) The active site of AcECH; (**C**) Docking Pose of AcECH with EC; (**D**) 2D diagram of bonding patterns between AcECH and EC.

Molecular docking is an effective method to analyze the binding modes of receptor and ligand molecules and to evaluate the binding capacity. It can be used in enzyme engineering to analyze the catalytic mechanism and compare the binding capacity of different ligand molecules. In this study, semi-flexible docking was employed to obtain more accurate and realistic binding modes. Specifically, based on the conservation analysis of the protein (Figure 2A) and the internal cavity structure of the protein, the active site (coordinates: −0.196228, −6.92843, −4.30248) was identified and the amino acids in this region were determined to be the flexible area. During the docking process, these areas were allowed to freely relax. This method better simulates the structural dynamics of the actual binding scenario. A total of 11 docking poses were generated. After excluding structurally impractical poses (e.g., due to amino acid-ligand overlaps or ligand deformation), the binding energies of the protein and ligand in the remaining docking poses were calculated, and the docking poses with the highest interaction energy were selected. The binding mode of the active site of AcECH with EC is shown in Figure 2B–D.

Various types of interactions were observed between AcECH and EC. Notably, SER97, THR98 and MET128 formed hydrogen bonds with the ester group and amino group of EC, respectively. Additionally, a series of hydrophobic interactions were identified, where residues PRO32, VAL124, PHE143, LEU146, LEU161, PHE166, TRP184, PHE203, VAL230 and HIS256 established van der Waals forces with EC, creating extensive hydrophobic interactions. Furthermore, TRP31 and ILE229 stabilized the substrate orientation through interaction forces such as π-alkyl interactions. Among these, hydrogen bonding provided the strongest binding energy, forming the predominant stabilizing force between EC and AcECH. Specifically, SER97 and THR98 in AcECH formed two hydrogen bonds with the ester group of EC, demonstrating AcECH’s high specificity for the ester group and reflecting its substrate-specific recognition ability as an esterase. The presence of these numerous weak interaction forces further stabilized the binding of EC to AcECH, facilitating efficient catalytic hydrolysis.

Analysis of sequence and structural conservation confirms that, among several highly conserved serine residues, SER97 acts as the core catalytic nucleophile of this enzyme. Three-dimensional structural analysis reveals that SER97 is precisely situated within the characteristic ‘nucleophilic elbow’ motif (Gly-X-Ser-X-Gly). This defining structural feature in the hydrolase places SER97 at the apex of an extremely acute β-turn, endowing it with a strong nucleophilic attack capability to efficiently hydrolyze the ester bond of ethyl carbamate (typically forming a catalytic triad with spatially adjacent histidine and aspartic acid). Furthermore, HIS256 and ASP227 are located within the catalytic pocket near SER97; their imidazole rings are within an appropriate interaction range of the hydroxyl group of SER97 and are highly conserved, with spatial distances all within 4.0 Å. This spatial arrangement is highly consistent with the typical SER–ASP–HIS catalytic triad of the α/β-hydrolase family. It is therefore inferred that SER97, ASP227 and HIS256 collectively constitute the catalytic center of AcECH. In this context, ASP227 enhances the proton transfer capacity of HIS256 by stabilizing its charge distribution, whilst HIS256 acts as a proton acceptor to activate the hydroxyl group of SER97, enabling it to form an alkoxy anion with enhanced nucleophilicity. This allows for a nucleophilic attack on the carbonyl carbon of ethyl carbamate, leading to the formation of an acylation intermediate and ultimately the hydrolysis of the substrate. This catalytic mechanism is highly consistent with that of previously reported members of the carboxylesterase and α/β-hydrolase families, further validating the central role of SER97 as a key catalytic residue and providing an important theoretical basis for the subsequent elucidation of enzymatic catalytic mechanisms and molecular engineering. Similar catalytic mechanisms have been proposed and experimentally validated in serine esterases and related hydrolases [23,24].

### 3.3. Molecular Dynamics Simulations of AcECH/EC Complexes

The molecular docking results illustrate a static and idealized state of the binding interactions between proteins and ligands. However, to more accurately simulate the real dynamic environment of protein-ligand complexes in solution and account for environmental factors such as temperature and pressure, the AcECH/EC complexes obtained from docking were further analyzed using MD simulations. The RMSD is a critical parameter used to evaluate the stability of protein-ligand complexes by examining fluctuations in protein conformation over time. Smaller and consistent RMSD values are indicative of more stable binding. Additionally, the RMSF captures the degree of fluctuation for individual amino acid residues during the simulation period, thereby reflecting the dynamic stability of specific regions within the protein.

**Figure 3:**
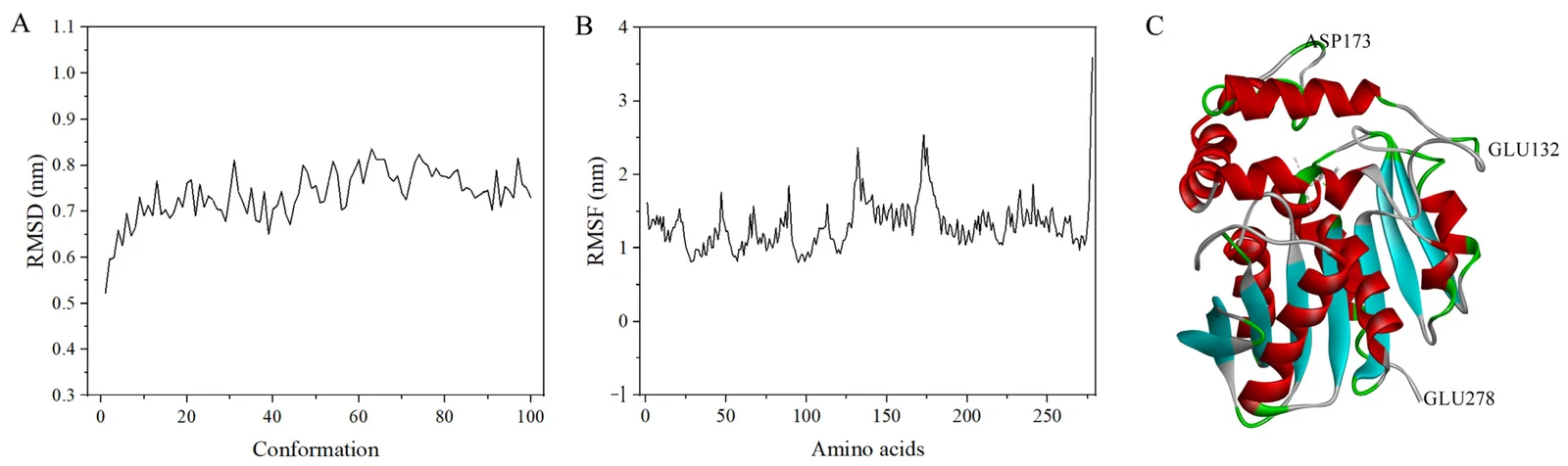
Molecular dynamics simulations of AcECH/EC complexes. (**A**) RMSD; (**B**) RMSF; (**C**) Unstable sites in the structure of AcECH/EC complexes.

As shown in Figure 3A, the RMSD values of the AcECH/EC complex demonstrate minimal fluctuations within a narrow range of 0.5–0.8 nm. This limited fluctuation indicates that the complex remains structurally stable throughout the simulation. The RMSF profile (Figure 3B) reveals that the majority of amino acid residues exhibit relatively low fluctuation, supporting the overall structural stability of the complex. However, certain localized regions exhibit higher RMSF values, signifying greater flexibility. Specifically, higher RMSF peaks were observed near residues GLU132, ASP173, and GLU278, with peak values of 2.36539 nm, 2.54236 nm, and 3.59176 nm, respectively. As illustrated in Figure 3C, these residues with elevated RMSF values are located in the outer irregular loop regions and at the terminus of the protein. These regions are inherently more flexible and active, as they are less structurally constrained compared to the core of the protein. This flexibility is characteristic of such regions and is often necessary for facilitating interactions with other molecules or accommodating structural adaptations during functional processes. The elevated RMSF values in these outer loop regions do not impact the global stability of the AcECH/EC complex, as evidenced by the consistent RMSD values.

### 3.4. GC-IMS Analysis of the Degradation Rate of Volatile Flavoring Substances by AcECH

Based on the above results, AcECH achieves efficient catalysis mainly by identifying the ester groups in the substrate and forming stable interactions with them. This is consistent with its esterase functional characteristics. Given that various volatile flavor compounds in Chinese Baijiu also contain ester structures, it is hypothesized that AcECH may also have certain catalytic activity for these flavor ester substances. Previous studies analyzed commercial Chinese Baijiu treated with AcECH, and the results showed that in addition to the target pollutant EC, important volatile ester substances such as ethyl acetate, ethyl hexanoate, and ethyl caprylate also underwent varying degrees of degradation [11]. Since these compounds are important components in the typical flavor of Chinese Baijiu, the action of this enzyme on the flavor quality of the liquor may have an adverse effect. Therefore, this study used gas chromatography-ion mobility spectrometry (GC-IMS) technology to systematically evaluate the degradation characteristics of these three esters by AcECH, and standard curves were generated by measuring the peak volumes obtained during GC-IMS analysis (Figure S2).

The degradation behavior of AcECH toward EC and representative volatile ethyl esters is summarized in Table 1. The EC degradation value (6.48%) was obtained from our previous study [11], whereas the degradation percentages of ethyl acetate, ethyl valerate, and ethyl hexanoate were determined experimentally in the present study using GC–IMS. Because EC and volatile ethyl esters were quantified using different analytical approaches and under different reaction conditions, these values should not be interpreted as directly comparable absolute degradation parameters. Instead, they were used as comparative indicators to characterize the substrate-dependent degradation behavior of AcECH.

The results demonstrated that, per milligram of AcECH, EC and ethyl acetate exhibited relatively high degradation percentages, reaching 6.48% and 29.69%, respectively, with minimal standard deviations. These findings indicate that AcECH possesses strong catalytic activity and excellent stability towards EC and ethyl acetate. In contrast, its degradation efficiency for ethyl valerate and ethyl hexanoate was markedly lower, at only 2.80% and 2.23%, respectively. This pronounced disparity suggests that AcECH exhibits a clear substrate specificity, with a strong catalytic preference for short-chain esters such as EC and ethyl acetate. Notably, these results are consistent with previous investigations into the enzymatic degradation of volatile flavor compounds in commercial Chinese Baijiu, which also indicated a similar pattern of catalytic selectivity across these four esters [11]. In both isolated systems and complex mixtures, AcECH consistently showed the highest catalytic efficiency toward ethyl acetate, slightly higher than its activity toward EC, followed by moderate activity for ethyl valerate and the lowest activity for ethyl hexanoate. These results indicate that AcECH is capable of recognizing and hydrolyzing multiple ester-containing substrates, with a relatively pronounced degradation of ethyl acetate among the volatile esters tested.

### 3.5. Molecular Basis of Substrate Specificity of AcECH Toward Volatile Ester Compounds in Chinese Baijiu

To investigate the catalytic mechanism and substrate specificity of AcECH toward representative volatile ester compounds in Chinese Baijiu, molecular docking analyses were performed using ethyl acetate, ethyl valerate, and ethyl hexanoate as representative flavor esters. EC was included as a reference substrate to analyze differences in binding conformations, interaction modes, and binding energies, providing support for subsequent engineering of AcECH substrate specificity.

**Figure 4:**
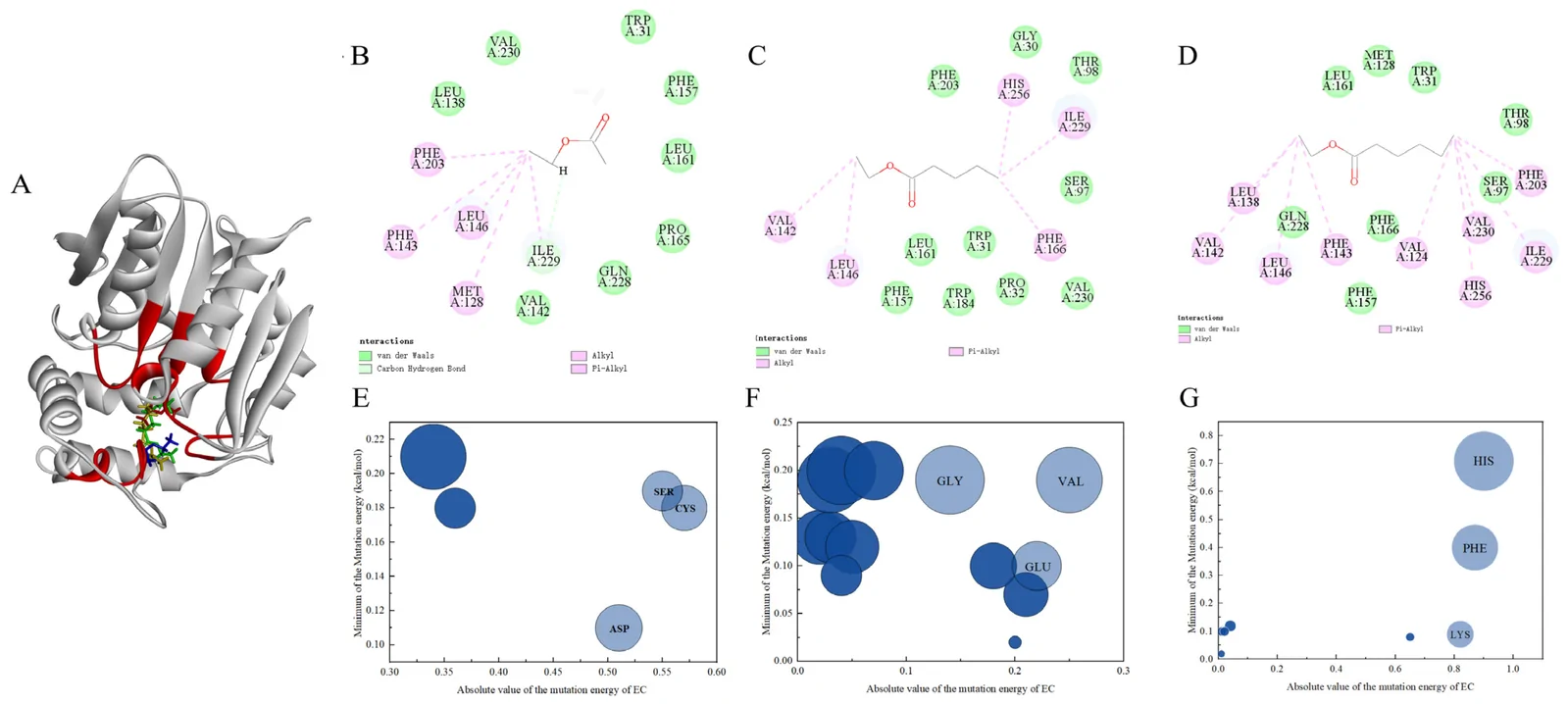
Molecular docking of AcECH with ethyl acetate, ethyl valerate and ethyl hexanoate. (**A**) Overlay diagram of binding sites of different ligands with AcECH. The red structure is EC, the blue structure is ethyl acetate, the yellow structure is ethyl valerate, and the green structure is ethyl hexanoate; (**B**) Bonding mode of AcECH binding to ethyl acetate; (**C**) Bonding mode of AcECH binding to ethyl valerate; (**D**) Bonding mode of AcECH binding to ethyl hexanoate. (**E**) Bubble plot of the mutation energies of the mutants at W31 with EC, ethyl acetate, ethyl valerate and ethyl hexanoate. The horizontal axis represents the absolute value of the binding energy with EC, while the vertical axis denotes the minimum value among the mutation energies for the three ethyl esters. Bubble size indicates the average binding energy with the three ethyl esters. (**F**,**G**) follow the same principle; (**F**) Bubble plot of the mutation energies of the mutants at F157 with EC, ethyl acetate, ethyl valerate and ethyl hexanoate; (**G**) Bubble plot of the mutation energies of the mutants at I229 with EC, ethyl acetate, ethyl valerate and ethyl hexanoate.

As shown in Table 2, AcECH exhibited the strongest binding energy toward ethyl acetate (−45.4259 kcal/mol), followed by EC (−41.5938 kcal/mol), ethyl valerate (−36.4749 kcal/mol), and ethyl hexanoate (−30.2122 kcal/mol). Figure 4A shows that the four substrates occupied similar binding positions within the AcECH active pocket. However, the larger molecular sizes and longer carbon chains of ethyl valerate and ethyl hexanoate introduced greater steric hindrance, which likely reduced substrate accessibility and catalytic efficiency.

As illustrated in Figure 4B–D, the interactions between AcECH and ester substrates were mainly mediated by non-covalent interactions, including van der Waals forces and π-alkyl interactions. Ethyl acetate formed a distinct C–H interaction with residue ILE229, which enhanced the stability of the complex compared with the other ester substrates. In contrast, the interactions between AcECH and ethyl valerate or ethyl hexanoate were dominated mainly by van der Waals interactions. For the AcECH–EC complex, hydrogen bonding was identified as the major interaction force. These different interaction modes directly contributed to the distinct binding energies and degradation efficiencies observed among the four substrates.

### 3.6. Identification of Key Residues and Mutant Design

Overall, AcECH exhibited the highest binding affinity toward ethyl acetate, followed by EC and ethyl valerate, whereas the weakest interaction was observed with ethyl hexanoate. This trend was highly consistent with the GC-IMS results, further supporting the reliability of the molecular docking analysis. Detailed examination of the docking poses revealed that residues TRP31 (W31), PHE157 (F157), and ILE229 (I229) were involved in interactions with volatile ethyl ester substrates but showed no direct interaction with EC. Given their potential role in discriminating between EC and competing ester substrates, these residues were considered promising targets for substrate-specificity engineering.

It can be learned from the conservative analysis in Figure 2A that although W31, F157, and I229 were found to be conserved among homologous sequences, structural analysis indicated that they were located within the substrate-binding region rather than the catalytic center. Combined with the docking results, these observations suggest that the conserved residues may contribute primarily to substrate recognition and binding. Therefore, targeted mutations at these positions were expected to alter interactions with competing ester substrates while minimizing disruption of EC degradation activity, thereby improving substrate specificity toward EC.

Virtual saturation mutation experiments were conducted on these key residues to evaluate the impact of changes in the key residues on the binding of different substrates. As shown in Figure 4E–G, the horizontal axis represents the absolute value of the mutagenic energy for the AcECH/EC complex, with higher values indicating stronger complex binding. The vertical axis denotes the lowest mutagenic energy among the three ethyl esters, where higher values signify a more pronounced reduction in the mutant’s binding capacity towards ethyl ester compounds. Therefore, the mutant shown in the figure has a reduced binding ability to all three ethyl esters, but its binding ability to EC remains stable or even increases, indicating a significant potential for specific optimization. According to the combined computational results, W31D, W31S, W31C, F157E, F157G, F157V, I229H, I229F, and I229K were selected for subsequent experimental verification, and the corresponding site-directed mutations were introduced into the wild-type enzyme. After electroporation, sequence confirmation, saturation mutation, transformation strain identification, expression, and purification, nine mutant enzymes were successfully obtained.

### 3.7. Characterization of Catalytic Properties of AcECH and Mutants

**Figure 5:**
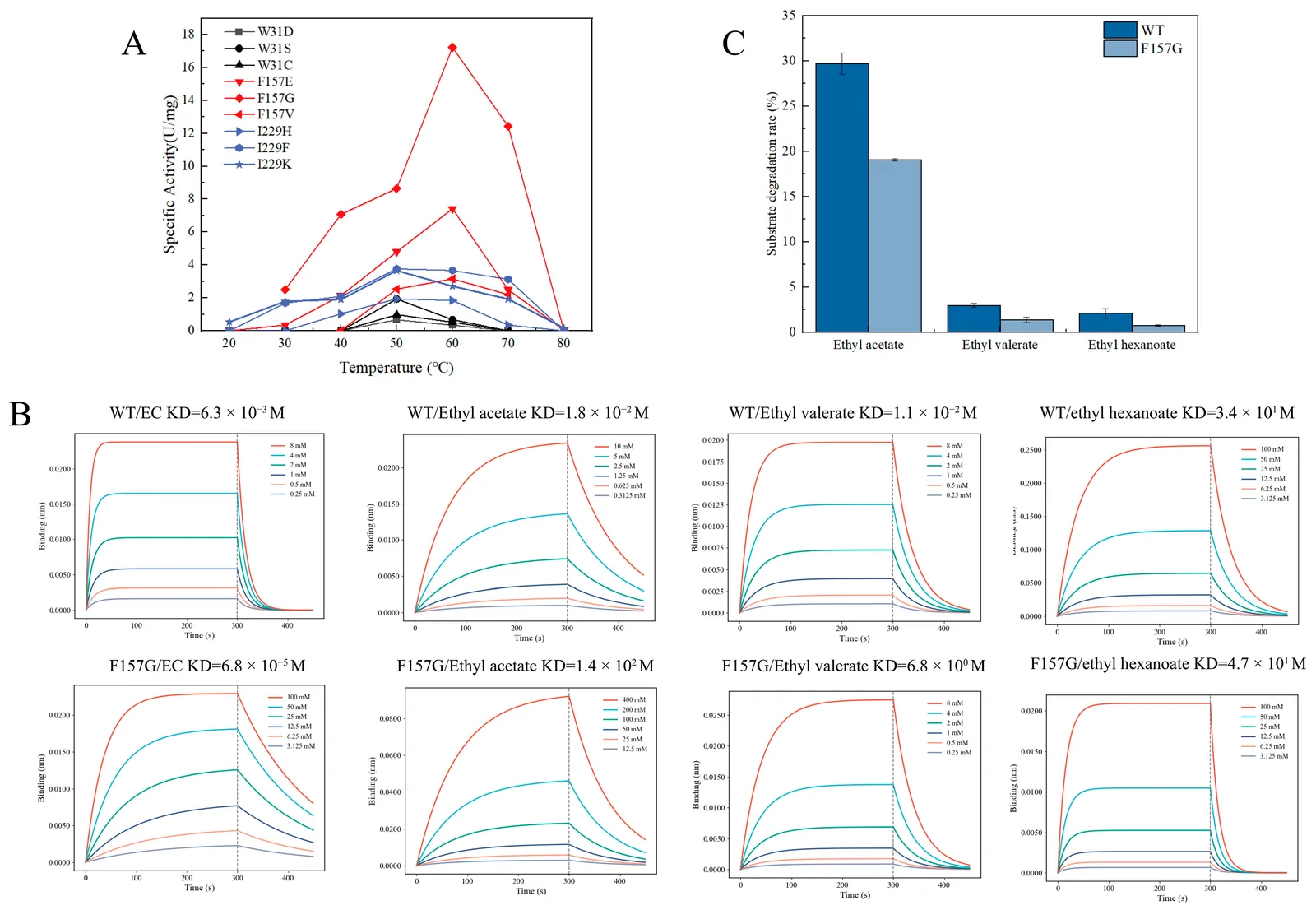
(**A**) Enzyme activity of the mutants and its variation with temperature; (**B**) The dissociation curves of AcECH and F157G respectively with EC, ethyl acetate, ethyl valerate and ethyl hexanoate; (**C**) The substrate degradation percentage of AcECH and the F157G for ethyl acetate, ethyl valerate and ethyl hexanoate.

The enzyme activities of each mutant at different temperatures are shown in Figure 5A. Compared with the wild-type enzyme, mutations at residues W31, I229, and F157 all resulted in varying degrees of reduced enzyme activity, among which the F157G mutant retained the highest residual activity, reaching 79.69% of wild-type activity. These results suggest that residues W31 and I229 may participate directly in EC binding and/or the catalytic process, whereas residue F157 appears to play a more regulatory role in substrate recognition rather than serving as an essential catalytic residue. The reduced activity observed in some mutants may result from local conformational changes or altered enzyme–substrate interactions caused by residue substitution.

To further evaluate the impact of the F157G mutation on the catalytic activity of EC, the kinetic parameters were determined using EC as the substrate. The results are shown in Table 3. Compared with the wild-type enzyme, the F157G mutant exhibited a significantly decreased apparent Km value toward EC, decreasing from 348.96 ± 75.81 mM to 154.48 ± 40.01 mM, indicating enhanced apparent affinity and improved substrate recognition toward EC. Meanwhile, the Vmax value decreased from 0.38 ± 0.04 μM/min to 0.27 ± 0.02 μM/min, suggesting a moderate reduction in the maximum catalytic rate. This result was consistent with the residual activity assay, in which F157G retained approximately 79.69% of the wild-type activity. Furthermore, the calculated kcat/Km value increased from 3.61 × 10^−6^ to 5.79 × 10^−6^ mM^−1^ s^−1^, indicating that the enhanced substrate recognition compensated for the slightly reduced catalytic turnover and resulted in improved overall catalytic efficiency toward EC.

These results suggest that replacement of Phe 157 with Gly did not impair EC utilization but instead optimized the balance between substrate binding and catalytic conversion. The decreased Vmax and kcat values may be attributed to subtle alterations in the local microenvironment of the substrate-binding pocket, which could influence the precise positioning of EC within the catalytic cavity and slightly affect the catalytic turnover process. However, the substantially reduced Km value indicates that F157G facilitated EC recognition and binding, ultimately leading to improved catalytic efficiency.

Considering that F157G simultaneously exhibited weakened binding affinity and reduced degradation capability toward volatile ethyl ester compounds, residue F157 is more likely involved in substrate recognition and pocket organization rather than directly participating in the catalytic mechanism of AcECH. Removal of the bulky aromatic side chain may remodel the hydrophobic microenvironment and spatial arrangement of the substrate-binding pocket, thereby reducing interactions with competing flavor-related esters while maintaining or enhancing the catalytic preference toward EC. In contrast, substitutions involving bulky or charged residues at positions W31 and I229 may have caused greater perturbation of the active-site architecture or substrate positioning, resulting in more pronounced activity loss.

### 3.8. Validation of Substrate Specificity by BLI and GC-IMS

To further investigate the effect of the F157G mutation on substrate recognition, BLI analysis was performed to characterize the binding interactions between AcECH and different ester substrates. As shown in Figure 5B, the F157G mutant exhibited a substantially enhanced binding affinity toward EC, with the KD value decreasing from 6.30 × 10^−3^ M to 6.80 × 10^−5^ M. In contrast, the KD values toward ethyl acetate, ethyl valerate, and ethyl hexanoate increased markedly after mutation, indicating significantly weakened interactions with volatile ethyl ester compounds. These results indicate that F157G reshaped the substrate-binding preference of AcECH, providing a favorable basis for selective EC recognition.

To further evaluate whether the altered binding behavior affected substrate degradation, GC-IMS analysis was performed to quantify the degradation percentages of representative volatile ester compounds. As shown in Figure 5C, the degradation percentage of ethyl acetate decreased from 29.69% to 19.07%, while the degradation percentages of ethyl valerate and ethyl hexanoate decreased from 2.80% to 1.38% and from 2.23% to 0.76%, respectively. The reduction in degradation efficiency was highly consistent with the BLI results, the enhanced binding affinity of F157G toward EC suggested that the mutation may facilitate substrate recognition; however, binding affinity alone does not directly represent catalytic efficiency. Combined with the reduced degradation of volatile esters observed by GC–IMS, these results suggest that F157G selectively redistributed substrate preference by enhancing EC recognition while reducing interactions with competing volatile esters.

Interestingly, despite the substantially enhanced binding affinity toward EC, the catalytic activity of the F157G mutant was moderately reduced. Although F157G exhibited a moderately decreased Vmax and kcat value compared with the wild-type enzyme, the substantially reduced Km value resulted in an increased catalytic efficiency (kcat/Km). This indicates that the mutation enhanced substrate recognition while causing only a minor influence on the catalytic turnover process. The reduced turnover rate may be attributed to subtle changes in substrate positioning or active-site microenvironment caused by the removal of the bulky aromatic side chain, rather than impaired substrate conversion. This observation suggests that enhanced substrate binding alone does not fully determine catalytic performance, as optimal catalytic efficiency requires a balance between substrate recognition and catalytic turnover.

In conclusion these findings indicate that residue F157 plays an important role in substrate recognition and specificity determination rather than directly participating in catalysis. The replacement of phenylalanine with glycine likely altered the topology and hydrophobic microenvironment of the substrate-binding pocket, thereby weakening hydrophobic interactions with volatile ethyl ester substrates while favoring EC recognition. Since phenylalanine contributes substantially to hydrophobic stabilization whereas glycine lacks a bulky hydrophobic side chain, the F157G mutation selectively reduced the affinity and degradation of flavor-related ester compounds while maintaining and even improving the catalytic efficiency toward EC. These results demonstrate that the F157G substitution altered the substrate-binding profile of AcECH, enhancing EC recognition preference while weakening the interaction with volatile ethyl ester substrates.

## 4. Conclusions

**Figure 6:**
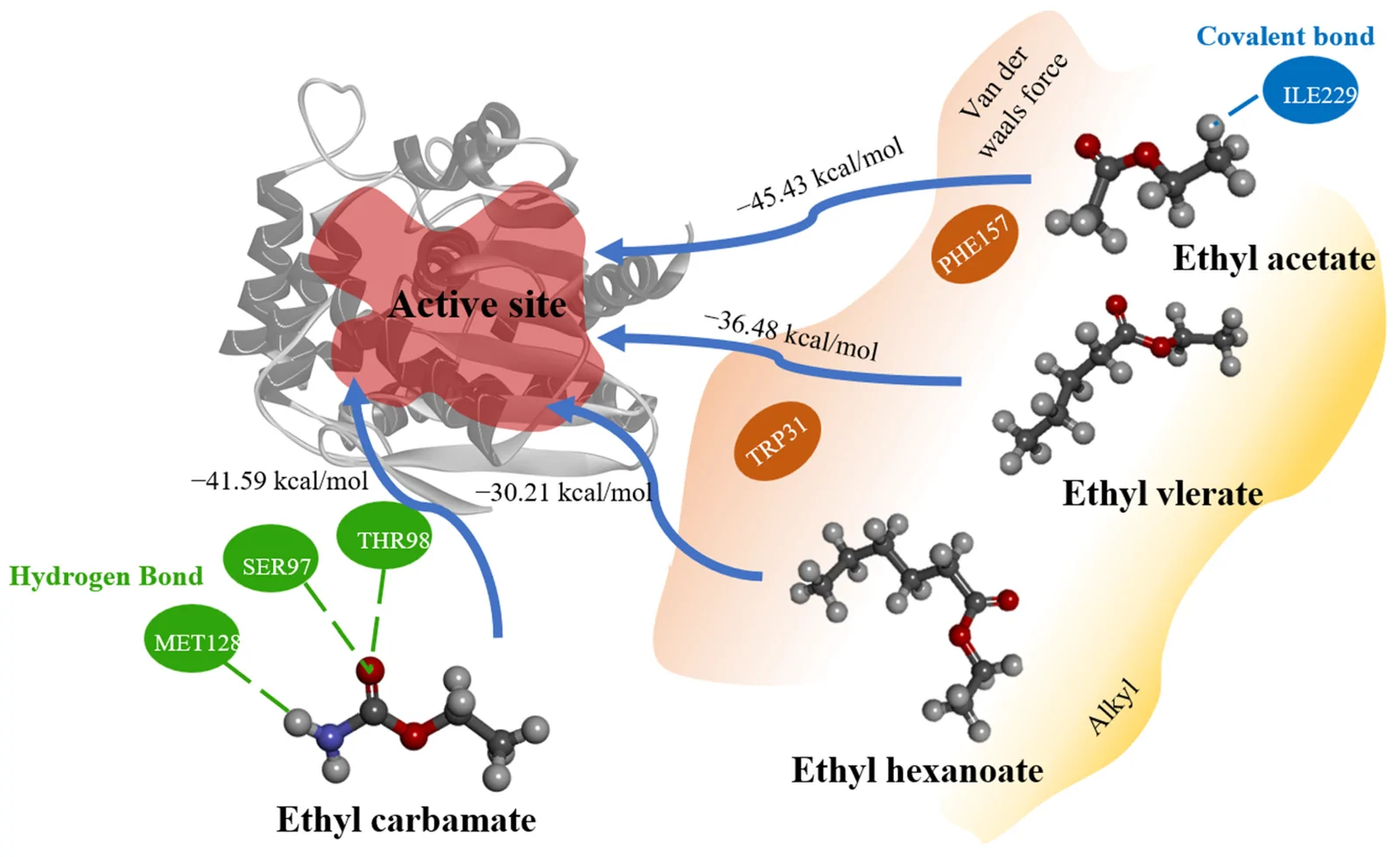
The mechanism of substrate specificity of AcECH.

This study investigated the molecular basis underlying the substrate preference of AcECH toward EC and volatile ethyl ester compounds through molecular simulation, kinetic analysis, bio-layer interferometry (BLI), and gas chromatography–ion mobility spectrometry (GC–IMS). Molecular analysis suggested that residues surrounding the substrate-binding region contribute to substrate discrimination by modulating the interaction preference of AcECH toward structurally different ester substrates. Among the selected residues, F157 showed a critical role in regulating substrate recognition, as mutation at this position caused distinct changes in the interaction profiles toward EC and volatile ethyl esters (Figure 6).

The F157G variant exhibited substantially enhanced binding affinity toward EC, with the KD value decreasing from 6.30 × 10^−3^ M to 6.80 × 10^−5^ M, while its interactions with ethyl acetate, ethyl valerate, and ethyl hexanoate were reduced. Consistently, GC–IMS analysis demonstrated decreased degradation of these volatile ethyl esters after F157 substitution. Although F157G exhibited a moderate decrease in Vmax, the substantially reduced Km value resulted in an increased kcat/Km value, suggesting that enhanced substrate recognition compensated for the slight reduction in catalytic turnover. These findings highlight the importance of balancing substrate binding and catalytic conversion during the engineering of EC-degrading enzymes.

Overall, this study provides a structural basis for modulating the substrate preference of AcECH through rational residue modification. Rather than solely enhancing EC degradation efficiency, the present strategy aims to improve the compatibility of EC-degrading enzymes with flavor-preserving requirements in fermented foods. The F157G variant provides a potential starting point for developing AcECH-based biocatalysts with reduced interference toward desirable flavor esters. Nevertheless, the current evaluation was performed mainly in model systems, and further studies are required to assess the performance of engineered AcECH in complex Baijiu matrices. Future studies should aim to achieve a better balance between EC recognition, catalytic turnover, and flavor preservation, while clarifying how structural modifications at this site affect substrate binding and enzymatic turnover.

## Figures and Tables

**Table 1:** Substrate degradation of EC, ethyl acetate, ethyl valerate and ethyl hexanoate by AcECH.

Substrate	Substrate Degradation Rate per Milligram of Enzyme (%)
Ethyl carbamate	6.48 *
Ethyl acetate	29.69 ± 0.98
Ethyl valerate	2.80 ± 2.26
Ethyl hexanoate	2.23 ± 1.66

* = Data source: [11].

**Table 2:** Binding energy of AcECH to EC, ethyl acetate, ethyl valerate and ethyl hexanoate.

Substrate	Binding Energy (kcal/mol)
Ethyl carbamate	−41.5938
Ethyl acetate	−45.4259
Ethyl valerate	−36.4749
Ethyl hexanoate	−30.2122

**Table 3:** Kinetic parameters of AcECH and F157G towards EC.

Substrate	Km (mM)	Vmax (μM/μmin)	kcat(s^−1^)	kcat/Km (mM^−1^s^−1^)	R^2^
AcECH	348.96 ± 75.81	0.38 ± 0.04	1.26 × 10^−3^	3.61 × 10^−6^	0.984
F157G	154.48 ± 40.01	0.27 ± 0.02	8.95 × 10^−4^	5.79 × 10^−6^	0.964

## Data Availability

The original contributions presented in this study are included in the article/Supplementary Materials. Further inquiries can be directed to the corresponding authors.

## Supplementary Materials

The following supporting information can be downloaded at: https://www.mdpi.com/article/10.3390/foods15173118/s1, Figure S1: Standard curve of NH4^+^.; Figure S2: Standard curve. A: Ethyl acetate; B: Ethyl valerate; C: Ethyl hexanoate; Table S1: Primers used for site-directed mutagenesis; Figure S3: Michaelis-Menten equation fitting curve. A: AcECH; B: F157G.
