# Supplementary material for: Substrate Recognition Analysis in an Ethyl Carbamate-Hydrolyzing Esterase and Semi-Rational Baijiu Flavor Preservation
Source: Foods. 2026 Sep 2;15(17):3118. doi: 10.3390/foods15173118 (PMC13565478; doi:10.3390/foods15173118)
Supplement: Supplementary file 1 [file foods-15-03118-s001.zip › foods-4506644-supplementary.pdf]

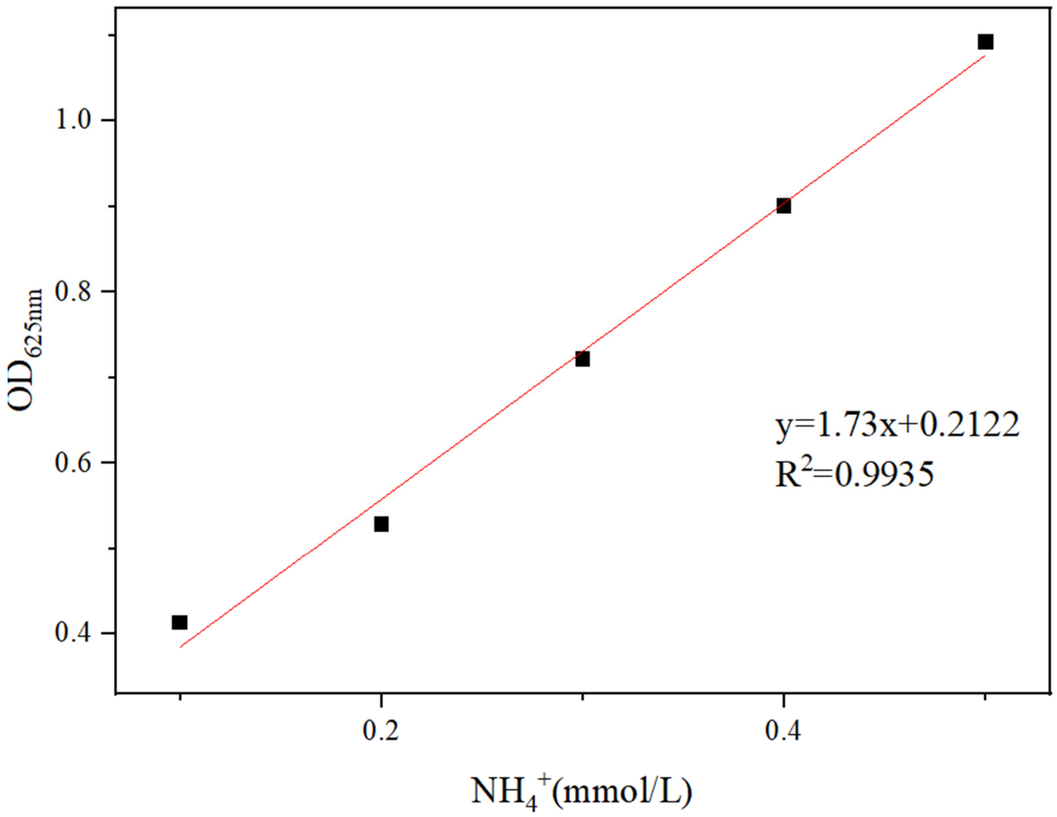

Figure S1. Standard curve of NH4$^+$.

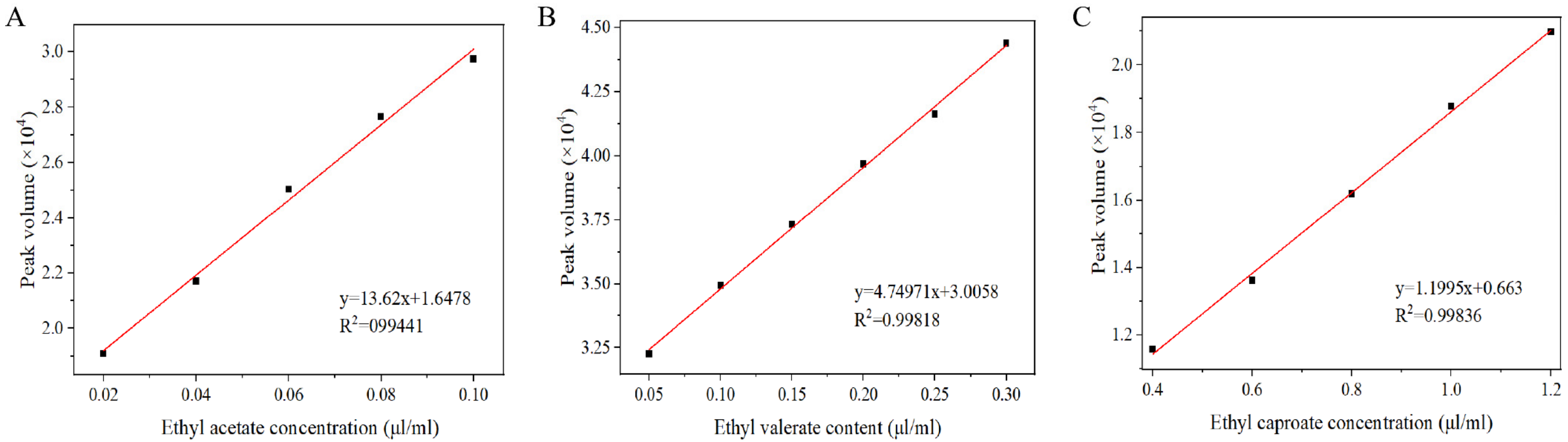

Figure S2. Standard curve. A: Ethyl acetate; B: Ethyl valerate; C: Ethyl hexanoate.

Table S1: Primers used for site-directed mutagenesis

Mutants	Mutagenic primers
W31D-F	5′-CGGATCGCCATGATGAAAAAACAGCACTTGCG-3′
W31D-R	5′-TTCATCATGGCGATCCGCTGAGCAGCGATGAT-3′
W31S-F	5′-TCAGCGGGCTGCCATGATGAAAAAACAGCACTTGCG-3′
W31S-R	5′-TTTCATCATGGCAGCCCGCTGAGCAGCGATGAT-3′
W31C-F	5′-TCAGCGGACAGCCATGATGAAAAAACAGCACTTGCG-3′
W31C-R	5′-TTTCATCATGGCTGTCCGCTGAGCAGCGATGAT-3′
F157E-F	5′-ATCGCGAAACTCCTGCGCGCGGTTGGTCAGCA-3′
F157E-R	5′-CGCAGGAGTTTCGCGATCTGCCGAGCGGCCCG-3′
F157G-F	5′-ATCGCGAAAGCCCTGCGCGCGGTTGGTCAGCA-3′
F157G-R	5′-CGCAGGGCTTTCGCGATCTGCCGAGCGGCCCG-3′
F157V-F	5′-ATCGCGAAACACCTGCGCGCGGTTGGTCAGCA-3′
F157V-R	5′-CGCAGGTGTTTCGCGATCTGCCGAGCGGCCCG-3′
I229H-F	5′-ACGGCACATGCTGATCATCATCGCCATGCAGC-3′
I229H-R	5′-TGATCAGCATGTGCCGTATAAAACGAGCGGCG-3′
I229F-F	5′-ACGGCACAAACTGATCATCATCGCCATGCAGC-3′
I229F-R	5′-TGATCAGTTTGTGCCGTATAAAACGAGCGGCG-3′
I229K-F	5′-ACGGCACTTTCTGATCATCATCGCCATGCAGC-3′
I229K-R	5′-TGATCAGAAAGTGCCGTATAAAACGAGCGGCG-3′

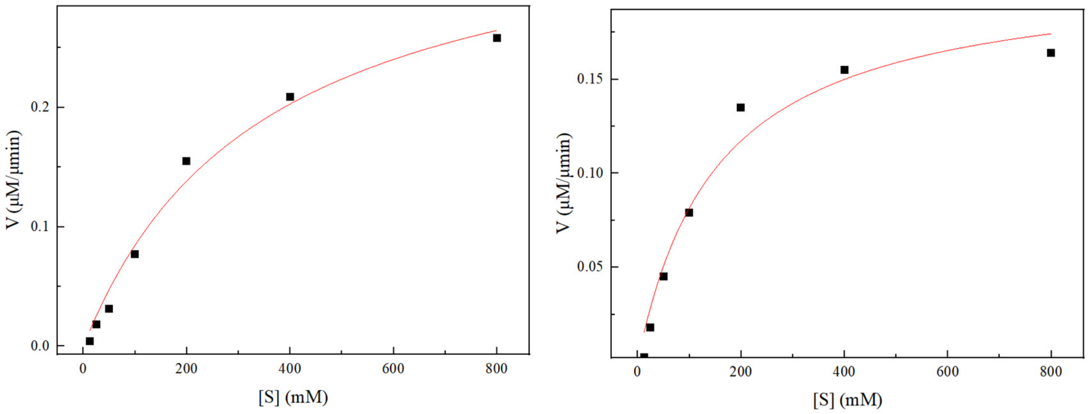

Figure S3. Michaelis-Menten equation fitting curve. A: AcECH; B: F157G.
